# Compound distributions for financial returns

**DOI:** 10.1371/journal.pone.0239652

**Published:** 2020-10-02

**Authors:** Emmanuel Afuecheta, Artur Semeyutin, Stephen Chan, Saralees Nadarajah, Diego Andrés Pérez Ruiz

**Affiliations:** 1 Department of Mathematics and Statistics, King Fahd University of Petroleum & Minerals, Dhahran, Saudi Arabia; 2 School of Economics, Finance and Accounting, Coventry University, Coventry, United Kingdom; 3 Department of Mathematics and Statistics, American University of Sharjah, Sharjah, UAE; 4 Department of Mathematics, University of Manchester, Manchester, United Kingdom; Universidad de Salamanca, SPAIN

## Abstract

In this paper, we propose six Student’s *t* based compound distributions where the scale parameter is randomized using functional forms of the half normal, Fréchet, Lomax, Burr III, inverse gamma and generalized gamma distributions. For each of the proposed distribution, we give expressions for the probability density function, cumulative distribution function, moments and characteristic function. GARCH models with innovations taken to follow the compound distributions are fitted to the data using the method of maximum likelihood. For the sample data considered, we see that all but two of the proposed distributions perform better than two popular distributions. Finally, we perform a simulation study to examine the accuracy of the best performing model.

## 1 Introduction

The Student’s *t* distribution due to Gosset [1] is the most common and parsimonious model for economic and financial data [2, 3]. It not only offers the potential to fit the leptokurtic properties of financial data but also, can serve as a foundation for building complex statistical models that can describe more subtle features of financial data such as volatility clustering. In recent times, many notable modifications to its functional form have been proposed, for example, see Hansen [4], Fernández and Steel [5], Theodossiou [6], Jones and Faddy [7], Sahu *et al.* [8], Bauwens and Laurent [9], Aas and Haff [10], Zhu and Galbraith [11, 12] and Papastathopoulos and Tawn [13]. They have been applied beyond Bayesian finite and infinite variance models [14], Markov regime switching models [15] as well as multivariate stochastic volatility models [16]. A detailed review of various modifications of the Student’s *t* distribution is provided by Li and Nadarajah [17] but the list is still by no means complete.

One of the Student’s *t* popular generalizations, often recommended for risk quantification in finance as noted by McNeil *et al.* [18] is the generalized hyperbolic distribution (GHYP) due to Barndorff-Nielsen [19]. The GHYP distribution offers a flexible functional form and possesses a number of attractive properties. For instance, the GHYP distribution can be both symmetric and skewed and is classified as a normal mean-variance mixture distribution and has the Student’s *t* as one of its special cases. Normal mean-variance distributions are also widespread and not uncommon. For example, mixing of this type can be traced back to Press [20] and Praetz [21], followed by Andrews and Mallows [22], Barndorff-Nielsen [19], Barndorff-Nielsen *et al.* [23], Kon [24], West [25], Madan and Seneta [26], Madan *et al.* [27], Tjetjep and Seneta [28], Luciano and Semeraro [29], Geweke and Amisano [30], Nadarajah [31], among others. Typically, these class of models (compound distributions) capture heterogeneous characteristics of financial data by randomizing one of the parameters (often the scale parameter) of the parent distribution with appropriate mixing distributions, for example, see McDonald and Butler [32], Hoogerheide *et al.* [33] and Ardia *et al.* [34, 35].

Recently, Afuecheta *et al.* [36], unlike the previous compositions which are based on the normal distribution, introduced mixture models based on scale mixing of the Student’s *t* distribution by specifically focusing on the leptokurtic properties of financial data. In particular, they provided flexible compositions of the Student’s *t* with three mixing distributions: exponential, Weibull and gamma. Their models were shown to provide better fits than some of the popular and more complicated generalizations of the Student’s *t* distribution, including the GHYP distribution. Hence, given good empirical performance of these models and because of the increasing interest in terms of methodology and applications, we extend this work by considering six mixing distributions. We proceed with the assumption that the conditional distribution for financial returns follows the Student’s t distribution. The variance (volatility) of returns is assumed to follow any of the six mixing distributions: one parameter half normal, two parameter Fréchet, two parameter Lomax, two parameter Burr III, two parameter inverse gamma and three parameter generalized gamma distributions. With these distributions, our research offers six new compound distributions.

The primary objectives of this paper are: (i) to propose six new compound distributions based on the Student’s *t* distribution; (ii) to illustrate applications of these distributions using real financial data sets; (iii) to compare the proposed distributions with two of the most popular parametric distributions used in finance–the GHYP distribution and asymmetric Student’s *t* (AST) distribution due to Zhu and Galbraith [11, 12]. For each of the proposed compound distribution, we provide its probability density function (PDF), cumulative distribution function (CDF), moments and characteristics functions. We perform our estimations using the method of maximum likelihood (ML). For the samples considered, empirical comparisons are made using a common set of log-likelihood based criteria. We show that all but one of the proposed distributions perform better than the GHYP distribution under the selection criteria. We also show that all but two of the proposed distributions perform better than the AST distribution under the selection criteria.

The rest of this paper is organized as follows. In Section 2 and corresponding subsections, we present the general form of the proposed distributions; Section 3 describes the data, conducts some exploratory analysis linked to the proposed distributions and outlines evaluation criteria; the results and their discussion are given in Section 4. In Section 5, we conduct a simulation study to assess the performance of the ML estimators with respect to sample size *n* and to demonstrate the ability of the best performing model. The simulation study also helps to evaluate the uncertainty surrounding the parameters of the best performing model, which ensures that the results obtained are reproducible if the same model is applied to the same data sets, but at a different time interval; finally, Section 6 concludes and summarises our work.

Two of the data sets used are data on cryptocurrencies. There are many papers on risk estimation for cryptocurrency data. Most notable papers include Acereda *et al.* [37], Trucios *et al.* [38] and Jimenez *et al.* [39].

## 2 Compound distributions

In this section, we begin by writing down the general form of the proposed distributions. Let *X* denote a continuous random variable representing the observed financial data series; in our case, log-returns of two financial stock indices, two fuel commodities and two cryptocurrencies exchange rates. Assuming that the conditional asset return distribution is Student’*t* with the PDF given by

$$\begin{array}{c} f(x|\sigma^{2})=\frac{\Gamma (\frac{\nu +1}{2})}{\sqrt{\nu \pi \sigma^{2}}\Gamma (\frac{\nu }{2})}{\left(1+\frac{x^{2}}{2\sigma^{2}}\right)}^{-\frac{\nu +1}{2}} \end{array}$$ (1)

for − ∞ < *x* < ∞ and where *σ*^2^ > 0.

Now, assuming that the variance *σ*^2^ itself is a random variable with PDF given by *g*(*σ*^2^), then the unconditional/actual stock return distribution will be given by the PDF

$$\begin{array}{c} f_{X}\left(x\right)=\int_{0}^{\infty }\frac{\Gamma (\frac{\nu +1}{2})}{\sqrt{\nu \pi \sigma^{2}}\Gamma (\frac{\nu }{2})}{\left(1+\frac{x^{2}}{\nu \sigma^{2}}\right)}^{-\frac{\nu +1}{2}}g(\sigma^{2})d\sigma^{2}, \end{array}$$ (2)

for convenience, we shall let *σ*^2^ = *τ* and rewrite the Eq (2) as

$$\begin{array}{ccc} f_{X}\left(x\right) & = & \frac{\Gamma (\frac{\nu +1}{2})}{\sqrt{\nu \pi }\Gamma (\frac{\nu }{2})}\int_{0}^{\infty }\frac{1}{\sqrt{\tau }}{\left(1+\frac{x^{2}}{\nu \tau }\right)}^{-\frac{\nu +1}{2}}g\left(\tau \right)d\tau \\ & = & \frac{\nu^{\frac{\nu }{2}}\Gamma (\frac{\nu +1}{2})}{x^{\nu +1}\sqrt{\pi }\Gamma (\frac{\nu }{2})}\int_{0}^{\infty }\tau^{\frac{\nu }{2}}{\left(1+\frac{\nu \tau }{x^{2}}\right)}^{-\frac{\nu +1}{2}}g\left(\tau \right)d\tau . \end{array}$$ (3)

By making use of the series expansion

$$1+z{)}^{-a}=\sum_{k=0}^{\infty }\left(\begin{array}{c} -a\\ k \end{array}\right)z^{k},$$

we can further simplify (3) as

$$\begin{array}{ccc} f_{X}(x) & = & \frac{\nu^{\frac{\nu }{2}}\Gamma (\frac{\nu +1}{2})}{x^{\nu +1}\sqrt{\pi }\Gamma (\frac{\nu }{2})}\sum_{k=0}^{\infty }(\begin{array}{l} \begin{array}{c} -\frac{\nu +1}{2}\\ k \end{array} \end{array})\frac{\nu^{k}}{x^{2k}}\int_{0}^{\infty }\tau^{k+\frac{\nu }{2}}g(\tau )d\tau \\ & = & \frac{\nu^{\frac{\nu }{2}}\Gamma (\frac{\nu +1}{2})}{x^{\nu +1}\sqrt{\pi }\Gamma (\frac{\nu }{2})}\sum_{k=0}^{\infty }{\left(\frac{\nu +1}{2}\right)}_{k}\frac{{\left(-\nu \right)}^{k}}{k!x^{2k}}\int_{0}^{\infty }\tau^{k+\frac{\nu }{2}}g(\tau )d\tau , \end{array}$$ (4)

where (*a*)_*k*_ = *a*(*a* + 1) ⋯ (*a* + *k* − 1) denotes the ascending factorial. Eq (4) is in its general form and shall be used to provide distributions for log-returns of our financial series. The general form of the CDF of *X* corresponding to (4) can be derived as

$$\begin{array}{c} F_{X}\left(x\right)=\frac{1}{2}+\frac{x\Gamma (\frac{\nu +1}{2})}{\sqrt{\pi \nu }\Gamma (\frac{\nu }{2})}\int_{0}^{\infty }\frac{1}{\sqrt{\tau }}\;{}_{2}F_{1}(\frac{1}{2},\frac{\nu +1}{2};\frac{3}{2};-\frac{x^{2}}{\tau \nu })g\left(\tau \right)d\tau \end{array}$$ (5)

for − ∞ < *x* < ∞. By making use of the series expansion

$$\begin{array}{ccc} {}_{2}F_{1}(a,b;c;z) & = & \frac{\Gamma \left(b-a\right)\Gamma \left(c\right){\left(-z\right)}^{-a}}{\Gamma (b)\Gamma (c-a)}\sum_{k=0}^{\infty }\frac{{\left(a\right)}_{k}{\left(a-c+1\right)}_{k}z^{-k}}{k!{\left(a-b+1\right)}_{k}}\\ & & \;\;\;+\frac{\Gamma \left(a-b\right)\Gamma \left(c\right){\left(-z\right)}^{-b}}{\Gamma (a)\Gamma (c-b)}\sum_{k=0}^{\infty }\frac{{\left(b\right)}_{k}{\left(b-c+1\right)}_{k}z^{-k}}{k!{\left(b-a+1\right)}_{k}}, \end{array}$$

we can further simplify (5) as

$$\begin{array}{c} F_{X}\left(x\right)=\frac{1}{2}-\frac{\nu^{\frac{\nu }{2}-1}\Gamma (\frac{\nu +1}{2})}{x^{\nu }\sqrt{\pi }\Gamma (\frac{\nu }{2})}\sum_{k=0}^{\infty }\frac{{\left(\frac{1+\nu }{2}\right)}_{k}{\left(\frac{\nu }{2}\right)}_{k}}{k!{\left(1+\frac{\nu }{2}\right)}_{k}}{\left(\frac{x^{2}}{\nu }\right)}^{-k}\int_{0}^{\infty }\tau^{k+\frac{\nu }{2}}g\left(\tau \right)d\tau . \end{array}$$ (6)

The general form of the *k*th moment of *X* can be expressed as

$$\begin{array}{c} E(X^{k})=E[E(X^{k}|\tau )]=[\int_{0}^{\infty }\tau^{\frac{k}{2}}g\left(\tau \right)d\tau ]\frac{1+{\left(-1\right)}^{k}}{2\sqrt{\pi }\Gamma (\frac{\nu }{2})}\Gamma (\frac{k+1}{2})\Gamma (\frac{\nu -k}{2})\nu^{\frac{k}{2}} \end{array}$$ (7)

provided that 0 < *k* < *ν*. The general form of the characteristic function of *X* can be expressed as

$$\begin{array}{c} E[\exp(itX)]=E\{E[\exp(itX)|\tau ]\}=[\int_{0}^{\infty }K_{\frac{\nu }{2}}(\sqrt{\nu \tau }|t|)\tau^{\frac{\nu }{4}}g\left(\tau \right)d\tau ]\frac{{\left(\sqrt{\nu }|t|\right)}^{\frac{\nu }{2}}}{2^{\frac{\nu }{2}-1}\Gamma (\frac{\nu }{2})}, \end{array}$$ (8)

where $i=\sqrt{-1}$ and *K*_*ν*_(⋅) denotes the modified Bessel function of the third kind defined by

$$\begin{array}{c} K_{n}\left(z\right)=\frac{\sqrt{\pi }x^{n}}{2^{n}\Gamma (n+\frac{1}{2})}\int_{1}^{\infty }\exp\left(-zt\right){\left(t^{2}-1\right)}^{n-\frac{1}{2}}dt. \end{array}$$

By making use of the series expansion

$$\begin{array}{c} K_{\nu }(z)=\frac{\pi \text{csc}(\pi \nu )}{2}[\sum_{k=0}^{\infty }\frac{z^{2k-\nu }}{2^{2k-\nu }k!\Gamma \left(k-\nu +1\right)}-\sum_{k=0}^{\infty }\frac{z^{2k+\nu }}{2^{2k+\nu }k!\Gamma \left(k+\nu +1\right)}], \end{array}$$

we can further simplify (8) as

$$\begin{array}{ccc} E[\exp(itX)] & = & \frac{{\left(\sqrt{\nu }|t|\right)}^{-\frac{\nu }{4}}\text{csc}(\frac{\pi \nu }{2})}{\Gamma (\frac{\nu }{2})}\sum_{k=0}^{\infty }\frac{{\left(\sqrt{\nu }|t|\right)}^{k}}{2^{2k}k!\Gamma (k-\frac{\nu }{2}+1)}[\int_{0}^{\infty }\tau^{k}g\left(\tau \right)d\tau ]\\ & & \;\;\;-\frac{{\left(\sqrt{\nu }|t|\right)}^{\frac{3\nu }{4}}\text{csc}(\frac{\pi \nu }{2})}{2^{\nu }\Gamma (\frac{\nu }{2})}\sum_{k=0}^{\infty }\frac{{\left(\sqrt{\nu }|t|\right)}^{k}}{2^{2k}k!\Gamma (k+\frac{\nu }{2}+1)}[\int_{0}^{\infty }\tau^{k+\frac{\nu }{2}}g\left(\tau \right)d\tau ]\\ & = & \frac{{\left(\sqrt{\nu }|t|\right)}^{-\frac{\nu }{4}}\text{csc}(\frac{\pi \nu }{2})}{\Gamma (\frac{\nu }{2})\Gamma (1-\frac{\nu }{2})}\sum_{k=0}^{\infty }\frac{{\left(\sqrt{\nu }|t|\right)}^{k}}{2^{2k}k!{\left(1-\frac{\nu }{2}\right)}_{k}}[\int_{0}^{\infty }\tau^{k}g\left(\tau \right)d\tau ]\\ & & \;\;-\frac{{\left(\sqrt{\nu }|t|\right)}^{\frac{3\nu }{4}}\text{csc}(\frac{\pi \nu }{2})}{2^{\nu }\Gamma (\frac{\nu }{2})\Gamma (1+\frac{\nu }{2})}\sum_{k=0}^{\infty }\frac{{\left(\sqrt{\nu }|t|\right)}^{k}}{2^{2k}k!{\left(\frac{\nu }{2}+1\right)}_{k}}[\int_{0}^{\infty }\tau^{k+\frac{\nu }{2}}g\left(\tau \right)d\tau ]. \end{array}$$ (9)

**Fig 1:**
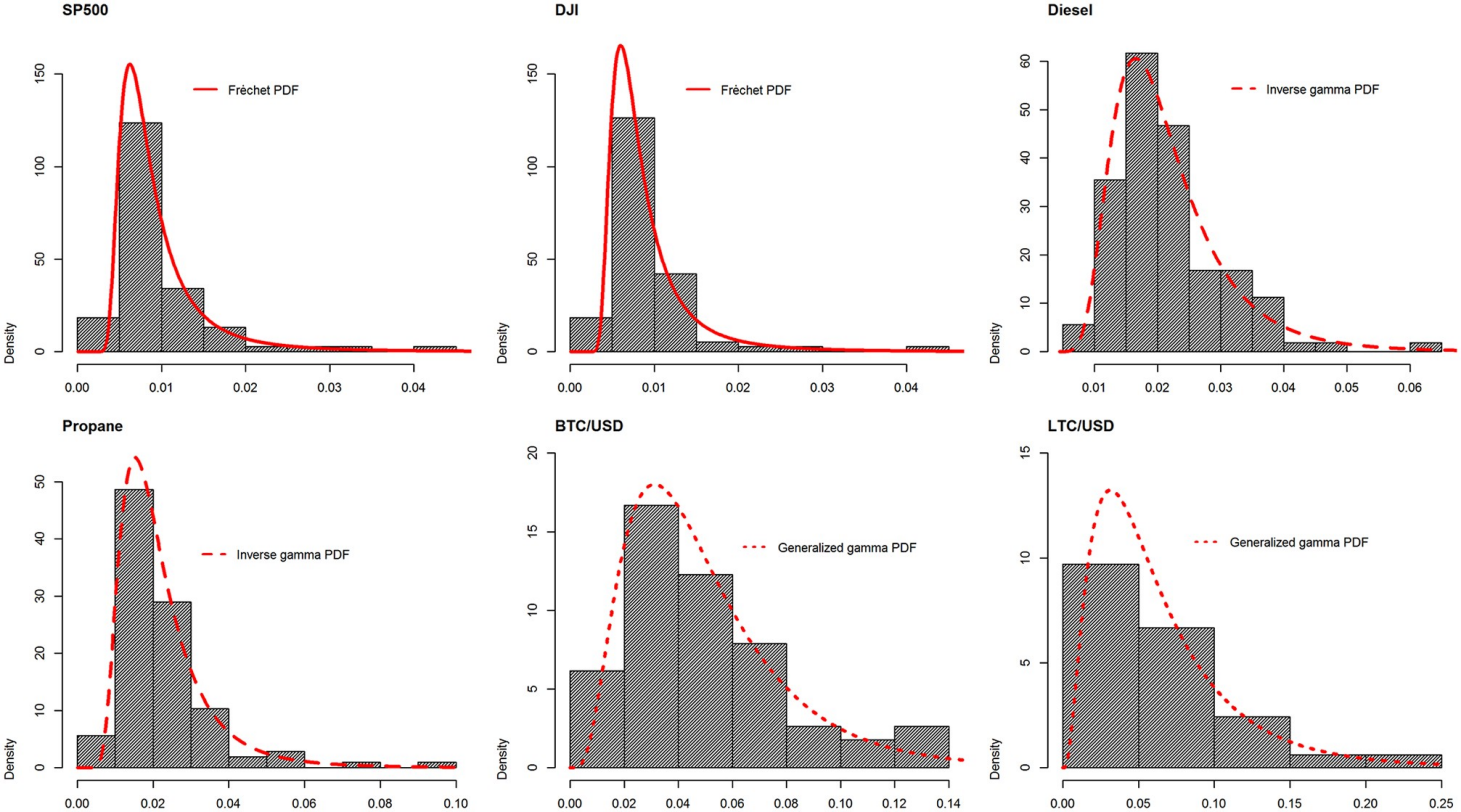
Histogram of standard deviations computed over non-overlapping windows of length 50 days for the specified daily log-returns (S&P500, DJI, Diesel, Propane, BTC and LTC).

Having obtained the general expressions for the PDF given by (4), the CDF given by (6), the *k*th moment given by (7), and the characteristic function given by (9), we shall proceed to obtain expressions for any given mixing distribution, *g*(⋅). The choice of the mixing distributions (two parameter inverse gamma distribution, two parameter Lomax distribution, the generalize gamma distribution, two parameter Burr distribution, two parameter Fréchet and one parameter half normal) is motivated by Fig 1, showing the histograms of the volatility for financial series considered in Section 3. The volatility is measured by the standard deviation taken over non-overlapping windows of length 50 days. From Fig 1, we see that *g*(⋅) corresponds to an exponential-type family of distributions with unimodal PDF, suggesting overall appropriateness of the choices. Notably, the following procedure was used for the choice of *g*(⋅): (i) fit the considered *g*(⋅) forms to the standard deviation series obtained using MLE; (ii) select the best performing *g*(⋅) based on the lowest negative log-likelihood and provide the best fitting parametric outcome for each histogram shown in Fig 1. With this, we observe that the volatility for stock indices and cryptocurrencies is best described by the generalized gamma PDF.

The calculations in the following sections make use of two special functions: the generalized hypergeometric function defined by

$$\begin{array}{c} {}_{p}F_{q}(a_{1},\ldots ,a_{p};b_{1},\ldots ,b_{q};x)=\sum_{k=0}^{\infty }\frac{{\left(a_{1}\right)}_{k}{\left(a_{2}\right)}_{k}\cdots {\left(a_{p}\right)}_{k}}{{\left(b_{1}\right)}_{k}{\left(b_{2}\right)}_{k}\cdots {\left(b_{q}\right)}_{k}}\frac{x^{k}}{k!}; \end{array}$$

the Wright [40] generalized hypergeometric function defined by

$$\begin{array}{c} {}_{p}\Psi_{q}[(\alpha_{1},A_{1}),\ldots ,(\alpha_{p},A_{p});(\beta_{1},B_{1}),\ldots ,(\beta_{q},B_{q});z]=\sum_{k=0}^{\infty }\frac{\prod_{j=1}^{p}\Gamma (\alpha_{j}+A_{j}k)}{\prod_{j=1}^{q}\Gamma (\beta_{j}+B_{j}k)}\frac{z^{k}}{k!}. \end{array}$$

The properties of these special functions can be found in Prudnikov *et al.* [41], Gradshteyn and Ryzhik [42], Mathai and Saxena [43] and Srivastava *et al.* [44].

### 2.1 Two parameter inverse gamma: With *g* taking the form

$$\begin{array}{c} g\left(\tau \right)=\frac{\beta^{\alpha }\tau^{-\alpha -1}\exp(-\frac{\beta }{\tau })}{\Gamma (\alpha )} \end{array}$$

for *τ* > 0, *α* > 0 and *β* > 0. Note that *β* and *α* are the scale and shape parameters, respectively. This PDF has a unique mode (which is found at *τ* = *β*/(*α*+ 1)) and skewed moderately to the right. It can be used to describe a wide range of physical phenomenon in diverse disciplines, including climatology, reliability, option pricing, economics, finance and survival analysis. See Bouchaud and Potters [45] for some application of the inverse gamma distribution to stock returns. For the two parameter inverse gamma distribution,

$$\begin{array}{c} \int_{0}^{\infty }\tau^{\eta }g\left(\tau \right)d\tau =\frac{\beta^{\eta }\Gamma \left(\alpha -\eta \right)}{\Gamma (\alpha )}. \end{array}$$

Hence, from (4), (6), (7), and (9) we obtain the closed form expressions for the PDF, CDF, moments and characteristic function as

$$\begin{array}{c} f_{X}\left(x\right)=\frac{{\left(\beta \nu \right)}^{\frac{\nu }{2}}\Gamma (\frac{\nu +1}{2})\Gamma (\alpha -\frac{\nu }{2})}{\Gamma (\alpha )x^{\nu +1}\sqrt{\pi }\Gamma (\frac{\nu }{2})}\;{}_{2}F_{0}(\frac{\nu +1}{2},\frac{\nu }{2}+1-\alpha ;;\frac{\nu \beta }{x^{2}}), \end{array}$$ (10)

$$\begin{array}{c} F_{X}\left(x\right)=\frac{1}{2}-\frac{\beta^{\frac{\nu }{2}}\nu^{\frac{\nu }{2}-1}\Gamma (\frac{\nu +1}{2})\Gamma (\alpha -\frac{\nu }{2})}{\Gamma (\alpha )x^{\nu }\sqrt{\pi }\Gamma (\frac{\nu }{2})}\;{}_{3}F_{1}(\frac{1+\nu }{2},\frac{\nu }{2},\frac{\nu }{2}+1-\alpha ;{\left(1+\frac{\nu }{2}\right)}_{k};-\frac{\nu \beta }{x^{2}}), \end{array}$$

$$\begin{array}{c} E(X^{k})=E[E(X^{k}|\tau )]=\frac{\beta^{\frac{k}{2}}\Gamma (-\frac{k}{2}+\alpha )}{\Gamma (\alpha )}\frac{1+{\left(-1\right)}^{k}}{2\sqrt{\pi }\Gamma (\frac{\nu }{2})}\Gamma (\frac{k+1}{2})\Gamma (\frac{\nu -k}{2})\nu^{\frac{k}{2}} \end{array}$$ (11)

and

$$\begin{array}{ccc} E[\exp(itX)] & = & \frac{{\left(\sqrt{\nu }|t|\right)}^{-\frac{\nu }{4}}\text{csc}(\frac{\pi \nu }{2})\Gamma \left(\alpha \right)}{\Gamma (\alpha )\Gamma (\frac{\nu }{2})\Gamma (1-\frac{\nu }{2})}\;{}_{1}F_{1}(1-\alpha ;1-\frac{\nu }{2};\frac{-\sqrt{\nu }\beta |t|}{4})\\ & & \;-\frac{\beta^{\frac{\nu }{2}}{\left(\sqrt{\nu }|t|\right)}^{\frac{3\nu }{4}}\text{csc}(\frac{\pi \nu }{2})\Gamma (\alpha -\frac{\nu }{2})}{\Gamma (\alpha )2^{\nu }\Gamma (\frac{\nu }{2})\Gamma (1+\frac{\nu }{2})}\;{}_{1}F_{1}(\frac{\nu }{2}+1-\alpha ;1+\frac{\nu }{2};\frac{-\sqrt{\nu }\beta |t|}{4}), \end{array}$$

respectively.

### 2.2 Two parameter Lomax: With *g* taking the form

$$\begin{array}{c} g\left(\tau \right)=\frac{\alpha \beta^{\alpha }}{{\left(\beta +\tau \right)}^{\alpha +1}} \end{array}$$

for *τ* > 0, *β* > 0 and *α* > 0. The scale and shape parameters are respectively governed by *β* and *α*. This PDF has a unique mode (with the mode at zero). It is notable for characterizing business failure. As a distribution within the Pareto family it has often used in modelling tail losses of returns. In fact, this distribution is also known as type II Pareto distribution and is a special case of the generalized Pareto. It has also been used extensively in analyzing lifetime data. See Benckert and Jung [46], Revankar *et al.* [47], Arnold [48], Hogg and Klugman [49] and Nair and Hitha [50] for some applications of the Lomax distribution. For the two parameter Lomax distribution,

$$\begin{array}{c} \int_{0}^{\infty }\tau^{\eta }g\left(\tau \right)d\tau =\frac{\beta^{\eta }\Gamma \left(\eta +1\right)\Gamma \left(\alpha -\eta \right)}{\Gamma (\alpha )}. \end{array}$$

Hence, from (4), (6), (7), and (9) we obtain the closed form expressions for the PDF, CDF, moments and characteristic function as

$$\begin{array}{c} f_{X}\left(x\right)=\frac{\alpha \beta^{\frac{\nu }{2}}\nu^{1+\frac{\nu }{2}}\Gamma (\frac{\nu +1}{2})\Gamma (\alpha -\frac{\nu }{2})}{2\sqrt{\pi }\Gamma (1+\alpha )x^{\nu +1}}\;{}_{3}F_{0}(\frac{\nu +1}{2},\alpha -\frac{\nu }{2},1+\frac{\nu }{2};;\frac{\nu \beta }{x^{2}}), \end{array}$$ (12)

$$\begin{array}{c} F_{X}\left(x\right)=\frac{1}{2}-\frac{\alpha \beta^{\frac{\nu }{2}}\nu^{\frac{\nu }{2}}\Gamma (\frac{\nu +1}{2})\Gamma (\alpha -\frac{\nu }{2})}{2\sqrt{\pi }\Gamma (1+\alpha )x^{\nu }}\;{}_{3}F_{0}(\frac{1+\nu }{2},\frac{\nu }{2},\frac{\nu }{2}+1-\alpha ;\frac{-\nu \beta }{x^{2}}), \end{array}$$

$$\begin{array}{c} E(X^{k})=E[E(X^{k}|\tau )]=\frac{\beta^{\frac{k}{2}}\Gamma (\frac{k}{2}+1)\Gamma (\alpha -\frac{k}{2})}{\Gamma (\alpha )}\frac{1+{\left(-1\right)}^{k}}{2\sqrt{\pi }\Gamma (\frac{\nu }{2})}\Gamma (\frac{k+1}{2})\Gamma (\frac{\nu -k}{2})\nu^{\frac{k}{2}} \end{array}$$ (13)

and

$$\begin{array}{ccc} E[\exp(itX)] & = & \frac{{\left(\sqrt{\nu }|t|\right)}^{-\frac{\nu }{4}}\text{csc}(\frac{\pi \nu }{2})}{\Gamma (\frac{\nu }{2})\Gamma (1-\frac{\nu }{2})}\;{}_{2}F_{1}(1,1-\alpha ;1-\frac{\nu }{2};-\frac{\sqrt{\nu }\beta |t|}{4})\\ & & \;-\frac{{\left(\sqrt{\nu }|t|\right)}^{\frac{3\nu }{4}}\text{csc}(\frac{\pi \nu }{2})\Gamma (\alpha -\frac{\nu }{2})}{2^{\nu }\Gamma (\frac{\nu }{2})}\;{}_{1}F_{0}(\frac{\nu }{2}+1-\alpha ;;-\frac{\sqrt{\nu }\beta |t|}{4}), \end{array}$$

respectively.

### 2.3 Generalized gamma: With *g* taking the form

$$\begin{array}{c} g\left(\tau \right)=\frac{\lambda \tau^{\alpha \lambda -1}\exp[-{\left(\frac{\tau }{\beta }\right)}^{\lambda }]}{\Gamma \left(\alpha \right)\beta^{\alpha \lambda }} \end{array}$$

for *τ* > 0, *β* > 0, λ > 0 and *α* > 0. The scale, first shape and second shape parameters are respectively given by *β*, λ and *α*. This PDF has a unique mode and skewed to the right. The generalized gamma distribution has extensive applications in different areas, including hydrology, water resources, biology, and economics. It encompasses a number of other distributions often used in survival analysis. For example, if λ = *α* = 1 then the generalized gamma distribution becomes the exponential distribution; if λ = 1 the generalized reduces to the gamma distribution; and if *α* = 1 the generalized becomes the Weibull distribution. For applications of this family of distribution to stock returns, see Madan and Seneta [26] and Tjetjep and Seneta [28]. For the generalized gamma distribution,

$$\begin{array}{c} \int_{0}^{\infty }\tau^{\eta }g\left(\tau \right)d\tau =\frac{\beta^{\eta }\Gamma (\alpha +\frac{\eta }{\lambda })}{\Gamma (\alpha )}. \end{array}$$

Hence, from (4), (6), (7), and (9) we obtain the closed form expressions for the PDF, CDF, moments and characteristic function as

$$\begin{array}{c} f_{X}\left(x\right)=\frac{\beta^{\frac{\nu }{2}}\nu^{\frac{\nu }{2}}}{\Gamma (\alpha )x^{\nu +1}\sqrt{\pi }\Gamma (\frac{\nu }{2})}\;{}_{2}\Psi_{0}[(\frac{\nu +1}{2},1),(\alpha +\frac{\nu }{2\lambda },\frac{1}{\lambda });;-\frac{\beta \nu }{x^{2}}], \end{array}$$ (14)

$$\begin{array}{c} F_{X}\left(x\right)=\frac{1}{2}-\frac{\beta^{\frac{\nu }{2}}\nu^{\frac{\nu }{2}}}{2\Gamma (\alpha )x^{\nu }\sqrt{\pi }\Gamma (\frac{\nu }{2})}\;{}_{3}\Psi_{1}[(\frac{\nu +1}{2},1),(\frac{\nu }{2},1),(\alpha +\frac{\nu }{2\lambda },\frac{1}{\lambda });(1+\frac{\nu }{2},1);\frac{\beta \nu }{x^{2}}], \end{array}$$

$$\begin{array}{c} E(X^{k})=E[E(X^{k}|\tau )]=\frac{\beta^{k/2}\Gamma (\frac{k}{2\lambda }+\alpha )}{\Gamma (\alpha )}\frac{1+{\left(-1\right)}^{k}}{2\sqrt{\pi }\Gamma (\frac{\nu }{2})}\Gamma (\frac{k+1}{2})\Gamma (\frac{\nu -k}{2})\nu^{\frac{k}{2}} \end{array}$$ (15)

and

$$\begin{array}{ccc} E[\exp(itX)] & = & \frac{{\left(\sqrt{\nu }|t|\right)}^{-\frac{\nu }{4}}\text{csc}(\frac{\pi \nu }{2})}{\Gamma (\alpha )\Gamma (\frac{\nu }{2})}\;{}_{1}\Psi_{1}[(\alpha ,\frac{1}{\lambda });(1-\frac{\nu }{2},1);\frac{\beta \sqrt{\nu }|t|}{4}]\\ & & \;-\frac{{\left(\sqrt{\nu }|t|\right)}^{\frac{3\nu }{4}}\beta^{\frac{\nu }{2}}\text{csc}(\frac{\pi \nu }{2})}{2^{\nu }\Gamma \left(\alpha \right)\Gamma (\frac{\nu }{2})}\;{}_{1}\Psi_{1}[(\alpha +\frac{\nu }{2\lambda },\frac{1}{\lambda });(1+\frac{\nu }{2},1);\frac{\beta \sqrt{\nu }|t|}{4}], \end{array}$$

respectively.

### 2.4 Two parameter Burr III distribution: With *g* taking the form

$$\begin{array}{c} g\left(\tau \right)=\frac{c\lambda \tau^{c-1}}{{\left(\tau^{c}+1\right)}^{\lambda +1}} \end{array}$$

for *τ* > 0, *c* > 0 and λ > 0. The two parameters are commonly referred to as the shape (*c*, λ) parameters. This distribution has a unique mode and moderately skewed to the right. The Burr distribution is one of the popular distribution in statistics. It is often used in reliability analysis as more flexible alternative to other competing distributions such as the lognormal, etc. It has a wide range of applications in other areas such as forestry, meteorology, etc. For the two parameter Burr III distribution,

$$\begin{array}{c} \int_{0}^{\infty }\tau^{\eta }g\left(\tau \right)d\tau =\frac{\Gamma (\frac{c+\eta }{c})\Gamma (\lambda -\frac{\eta }{c})}{\Gamma (\lambda )}. \end{array}$$

Hence, from (4), (6), (7), and (9) we obtain the closed form expressions for the PDF, CDF, moments and characteristic function as

$$\begin{array}{c} f_{X}\left(x\right)=\frac{\nu^{\frac{\nu }{2}}}{\Gamma \left(\lambda \right)x^{\nu +1}\sqrt{\pi }\Gamma (\frac{\nu }{2})}\;{}_{3}\Psi_{0}[(\frac{\nu +1}{2},1),(1+\frac{\nu }{2c},\frac{1}{c}),(\lambda -\frac{\nu }{2c},-\frac{1}{c});;-\frac{\nu }{x^{2}}], \end{array}$$ (16)

$$\begin{array}{c} F_{X}\left(x\right)=\frac{1}{2}-\frac{\nu^{\frac{\nu }{2}}}{2\Gamma \left(\lambda \right)x^{\nu }\sqrt{\pi }\Gamma (\frac{\nu }{2})}\;{}_{4}\Psi_{1}[(\frac{\nu +1}{2},1),(\frac{\nu }{2},1),(1+\frac{\nu }{2c},\frac{1}{c}),(\lambda -\frac{\nu }{2c},-\frac{1}{c});(1+\frac{\nu }{2},1);\frac{\nu }{x^{2}}], \end{array}$$

$$\begin{array}{c} E(X^{k})=E[E(X^{k}|\tau )]=\frac{\Gamma (\frac{k}{2c}+1)\Gamma (\lambda -\frac{k}{2c})}{\Gamma (\lambda )}\frac{1+{\left(-1\right)}^{k}}{2\sqrt{\pi }\Gamma (\frac{\nu }{2})}\Gamma (\frac{k+1}{2})\Gamma (\frac{\nu -k}{2})\nu^{\frac{k}{2}} \end{array}$$ (17)

and

$$\begin{array}{ccc} E[\exp(itX)] & = & \frac{{\left(\sqrt{\nu }|t|\right)}^{-\frac{\nu }{4}}\text{csc}(\frac{\pi \nu }{2})}{\Gamma (\lambda )\Gamma (\frac{\nu }{2})}\;{}_{2}\Psi_{1}[(1,\frac{1}{c}),(\lambda ,-\frac{1}{c});(1-\frac{\nu }{2},1);\frac{\sqrt{\nu }|t|}{4}]\\ & & \;-\frac{{\left(\sqrt{\nu }|t|\right)}^{\frac{3\nu }{4}}\text{csc}(\frac{\pi \nu }{2})}{\Gamma (\lambda )2^{\nu }\Gamma (\frac{\nu }{2})}\;{}_{2}\Psi_{1}[(1+\frac{\nu }{2c},\frac{1}{c}),(\lambda -\frac{\nu }{2c},-\frac{1}{c});(1+\frac{\nu }{2},1);\frac{\sqrt{\nu }|t|}{4}], \end{array}$$

respectively.

### 2.5 Two parameter Fréchet: With *g* taking the form

$$\begin{array}{c} g\left(\tau \right)=\frac{\alpha \beta^{\alpha }\exp[-{\left(\frac{\beta }{\tau }\right)}^{\alpha }]}{\tau^{\alpha +1}} \end{array}$$

for *τ* > 0, *α* > 0 and *β* > 0. This distribution has a unique mode and skewed to the right. The shape and scale parameters are, respectively, governed by *α* and *β*. The distribution is also known as inverse Weibull distribution because if 1/*Ω* has the Weibull distribution then *Ω* will have the Fréchet distribution. It is a special case of the generalized extreme value distribution which is widely used in characterization of “tail risks” in fields ranging from insurance to finance. Some other application areas of the Fréchet distribution include business and operations research, economics, hydrology, materials and product technology. For the two parameter Fréchet distribution,

$$\begin{array}{c} \int_{0}^{\infty }\tau^{\eta }g\left(\tau \right)d\tau =\beta^{\eta }\Gamma (1-\frac{\eta }{\alpha }). \end{array}$$

Hence, from (4), (6), (7), and (9) we obtain the closed form expressions for the PDF, CDF, moments and characteristic function as

$$\begin{array}{c} f_{X}\left(x\right)=\frac{{\left(\beta \nu \right)}^{\frac{\nu }{2}}}{x^{\nu +1}\sqrt{\pi }\Gamma (\frac{\nu }{2})}\;{}_{2}\Psi_{0}[(\frac{\nu +1}{2},1),(1-\frac{\nu }{2\alpha },-\frac{1}{\alpha });;-\frac{\beta \nu }{x^{2}}], \end{array}$$ (18)

$$\begin{array}{c} F_{X}\left(x\right)=\frac{1}{2}-\frac{{\left(\beta \nu \right)}^{\frac{\nu }{2}}}{2x^{\nu }\sqrt{\pi }\Gamma (\frac{\nu }{2})}\;{}_{3}\Psi_{1}[(\frac{\nu +1}{2},1),(\frac{\nu }{2},1),(1-\frac{\nu }{2\alpha },-\frac{1}{\alpha });(1+\frac{\nu }{2},1);\frac{\beta \nu }{x^{2}}], \end{array}$$

$$\begin{array}{c} E(X^{k})=E[E(X^{k}|\tau )]=\Gamma (1-\frac{k}{2\alpha })\frac{1+{\left(-1\right)}^{k}}{2\sqrt{\pi }\Gamma (\frac{\nu }{2})}\Gamma (\frac{k+1}{2})\Gamma (\frac{\nu -k}{2}){\left(\beta \nu \right)}^{\frac{k}{2}} \end{array}$$ (19)

and

$$\begin{array}{ccc} E[\exp(itX)] & = & \frac{{\left(\sqrt{\nu }|t|\right)}^{-\frac{\nu }{4}}\text{csc}(\frac{\pi \nu }{2})}{\Gamma (\frac{\nu }{2})}\;{}_{1}\Psi_{1}[(1,-\frac{1}{\alpha });(1-\frac{\nu }{2},1);\frac{\beta \sqrt{\nu }|t|}{4}]\\ & & \;-\frac{\beta^{\frac{\nu }{2}}{\left(\sqrt{\nu }|t|\right)}^{\frac{3\nu }{4}}\text{csc}(\frac{\pi \nu }{2})}{2^{\nu }\Gamma (\frac{\nu }{2})}\;{}_{1}\Psi_{1}[(1-\frac{\nu }{2\alpha },-\frac{1}{\alpha });(1+\frac{\nu }{2},1);\frac{\beta \sqrt{\nu }|t|}{4}], \end{array}$$

respectively.

### 2.6 One parameter half normal: With *g* taking the form

$$\begin{array}{c} g\left(\tau \right)=\frac{2}{\sqrt{2\pi }\theta }\exp(-\frac{\tau^{2}}{2\theta^{2}}) \end{array}$$

for *τ* > 0 and *θ* > 0. The half normal distribution is a normal distribution with scale parameter *θ* bounded from below at zero. Its applications cut across many areas. For instance, see Meeusen and van Den Broeck [51] and Chou and Liu [52] for applications of the half normal distribution in production processes; Lawless [53] and Cooray and Ananda [54] for applications in life data analysis; Dobzhansky and Wright [55] for applications in genetics; and Bland and Altman [56] for applications in biological sciences. For the one parameter half normal distribution,

$$\begin{array}{c} \int_{0}^{\infty }\tau^{\eta }g\left(\tau \right)d\tau =\frac{{\left(\sqrt{2}\theta \right)}^{\eta }}{\sqrt{\pi }}\Gamma (\frac{\eta +1}{2}). \end{array}$$

Hence, from (4), (6), (7), and (9) we obtain the closed form expressions for the PDF, CDF, moments and characteristic function as

$$\begin{array}{c} f_{X}\left(x\right)=\frac{{\left(\sqrt{2}\theta \nu \right)}^{\frac{\nu }{2}}}{x^{\nu +1}\pi \Gamma (\frac{\nu }{2})}\;{}_{2}\Psi_{0}[(\frac{\nu +1}{2},1),(\frac{\nu +2}{4},\frac{1}{2});;-\frac{\sqrt{2}\theta \nu }{x^{2}}], \end{array}$$ (20)

$$\begin{array}{c} F_{X}\left(x\right)=\frac{1}{2}-\frac{{\left(\sqrt{2}\theta \nu \right)}^{\frac{\nu }{2}}}{2x^{\nu }\pi \Gamma (\frac{\nu }{2})}\;{}_{3}\Psi_{1}[(\frac{\nu +1}{2},1),(\frac{\nu }{2},1),(\frac{\nu +2}{4},\frac{1}{2});(1+\frac{\nu }{2},1);\frac{\sqrt{2}\theta \nu }{x^{2}}], \end{array}$$

$$\begin{array}{c} E(X^{k})=E[E(X^{k}|\tau )]={\left(\sqrt{2}\theta \right)}^{k/2}\Gamma (\frac{k+2}{4})\frac{1+{\left(-1\right)}^{k}}{2\pi \Gamma (\frac{\nu }{2})}\Gamma (\frac{k+1}{2})\Gamma (\frac{\nu -k}{2})\nu^{\frac{k}{2}} \end{array}$$ (21)

and

$$\begin{array}{ccc} E[\exp(itX)] & = & \frac{{\left(\sqrt{\nu }|t|\right)}^{-\frac{\nu }{4}}\text{csc}(\frac{\pi \nu }{2})}{\sqrt{\pi }\Gamma (\frac{\nu }{2})}\;{}_{1}\Psi_{1}[(\frac{1}{2},\frac{1}{2});(1-\frac{\nu }{2},1);\frac{\sqrt{2\nu }\theta |t|}{4}]\\ & & \;-\frac{\theta^{\frac{\nu }{2}}{\left(\sqrt{\nu }|t|\right)}^{\frac{3\nu }{4}}\text{csc}(\frac{\pi \nu }{2})}{\sqrt{\pi }2^{\frac{3\nu }{4}}\Gamma (\frac{\nu }{2})}\;{}_{1}\Psi_{1}[(\frac{\nu +2}{4},\frac{1}{2});(1+\frac{\nu }{2},1);\frac{\sqrt{2\nu }\theta |t|}{4}], \end{array}$$

respectively.

### 2.7 Skewness and kurtosis

**Fig 2:**
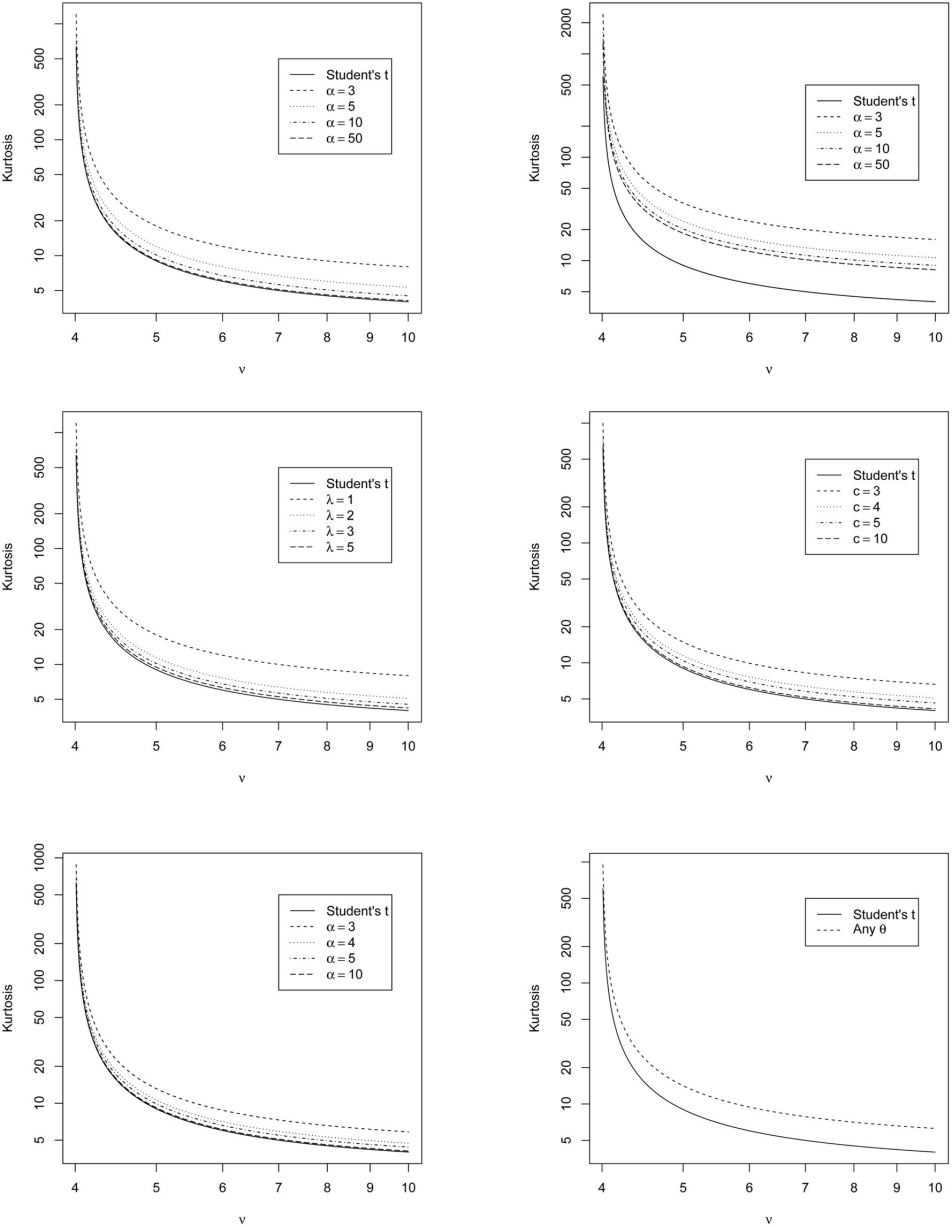
Kurtosis values corresponding to (11) (top left), (13) (top right), (15) (middle left), (17) (middle right), (19) (bottom left) and (21) (bottom right) versus *ν* and selected values of other parameters.

By definition, each of the six compound distributions has zero skewness. The kurtosis values can be computed using (11), (13), (15), (17), (19) and (21). These values versus the degree of freedom parameter, *ν*, are shown in Fig 2.

We see that kurtosis is a decreasing function of *ν* for each compound distribution. The kurtosis for each distribution takes larger values compared to the Student’s *t* distribution; hence, they are more flexible with respect to heavy tailed data. Over the plotted range, the compound Lomax distribution takes the largest kurtosis values. We note further that the kurtosis is a decreasing function of: *α* for the compound inverse gamma distribution; *α* for the compound Lomax distribution; λ for the compound generalized gamma distribution; *c* for the compound Burr distribution; *α* for the compound Fréchet distribution.

For details about how skewness and kurtosis can be used to improve model fitting and forecasting performance, see Feunou *et al.* [57] and Lalancette and Simonato [58].

## 3 Data

**Fig 3:**
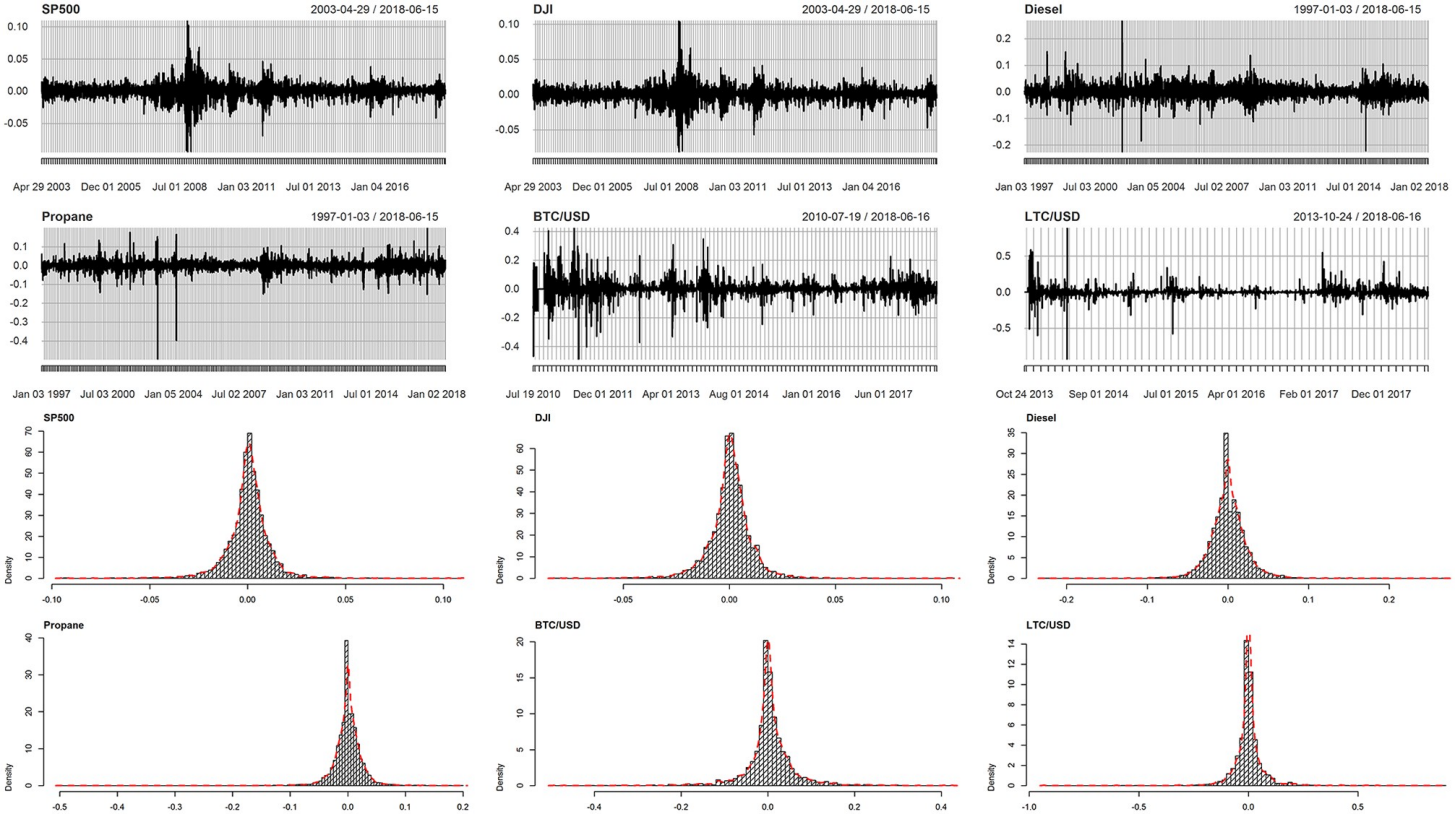
Time series plots of the daily log-returns of S&P500, DJI, Diesel, Propane, BTC and LTC with their histograms and kernel based density estimates.

To investigate the empirical performance of the proposed distributions, we consider six popular financial series. These include: two financial stock indices, two fuel commodities prices and two cryptocurrencies exchange rates. Stock indices are Standard & Poor’s 500 (S&P500) and Dow Jones Industrial Average (DJI) for the period starting from the 28th of April 2003 to the 15th of June 2018 as provided by Bloomberg. Fuel commodities are spot prices for the Los Angeles Ultra-Low-Sulfur Diesel (Diesel) and Mont Belvieu, Texas Propane (Propane) in USDs per gallon for the period starting from the 2nd of January 1997 to the 15th of June 2018 as provided by the United States Energy Information Administration. Cryptocurrencies are Bitcoin (BTC) for the period starting from the 18th of July 2010 to the 16th of June 2018 and Litecoin (LTC) for the period starting from the 24th of October 2013 to the 16th of June 2018. Both cryptocurrencies are denominated in USD with their sample sizes representing their entire life cycle on the moment of the data downloaded from Quandl, BNC2 database. For more extensive discussion on cryptocurrencies see Chan *et al.* [59] and the references therein. In general, there are no specific rationale for composing our data set, though alongside some very common stock indices (S&P500 and DJI) we aim to have some financial series with notable tail (excess kurtosis) characteristics (Propane and LTC), since our work is partially motivated by the heavy tail potential of the parent distribution of the compound distributions. For the above discussed financial series, we computed log-returns as

$$\begin{array}{c} R_{i,t}=\log(\frac{P_{i,t}}{P_{i,t-1}}), \end{array}$$

where *R*_*i*,*t*_ is the return on the index *i* for the period *t*, *P*_*i*,*t*_ is the closing rate/price of the index at the end of period *t* and *P*_*i*,*t*−1_ is the price of the index at the end of the period *t* − 1. The histogram of the transformed data and their kernel density evaluations are shown in Fig 3. Their characteristics described in Table 1 are: minimum, first quartile (Q1), median, mean, third quartile (Q3), maximum, skewness, kurtosis, standard deviation (SD), variance, range and inter quartile range (IQR).

**Table 1:** Summary statistics of daily log-returns of S&P500, DJI, Diesel, Propane, BTC and LTC.

Statistic	S&P500	DJI	Diesel	Propane	BTC	LTC
*n*	3811	3811	5388	5388	2890	1696
Minimum	-0.09469	-0.08201	-0.22716	-0.49913	-0.49152	-0.93452
Q1	-0.03903	-0.00388	-0.01149	-0.01025	-0.01273	-0.02003
Median	0.00069	0.00053	0.00000	0.00000	0.00141	0.00000
Mean	0.00029	0.00028	0.00019	0.00009	0.00386	0.00204
Q3	0.00526	0.00507	0.01139	0.01121	0.02276	0.01810
Maximum	0.10957	0.10508	0.26826	0.19979	0.42457	0.89035
Skewness	-0.37707	-0.15289	0.10768	-2.02435	-0.34656	0.63046
Kurtosis	15.2352	14.59654	13.8692	45.13415	14.70389	36.08584
SD	0.01146	0.01064	0.02336	0.02572	0.05840	0.07981
Variance	0.00013	0.00011	0.00054	0.00066	0.00341	0.00637
Range	0.20426	0.18708	0.49542	0.69892	0.91611	1.82488
IQR	0.00916	0.00895	0.02288	0.02146	0.03550	0.03814

From Table 1, we observe the highest range is for the cryptocurrencies returns, followed by the commodities and the smallest for the stock indices. Fig 3 shows the time series plots of returns which appear to oscillate around zero. The oscillations vary a great deal in magnitude, but are almost constant in average over period of the study. Also, from the plot we observe that for each return, periods of high volatility are followed by the periods of low volatility and vice versa. This is not surprising as it is a typical nature of financial indices [60–62]. Notably, from Fig 3, there is evidence of sharp market corrections for Diesel and Propane in the early 2000s. This could be explained by the changes in the fundamentals of hydrocarbons, while lack of the clearly defined fundamentals best explains the highest range for the cryptocurrencies. For the stock indices, the pronounced spikes around 2008 could be attributed to the events of the financial crisis, while their lowest range may be explained by their composite nature. The highest kurtosis values are depicted by the Propane and LTC. For S&P500, DJI, Deisel and BTC, the kurtosis values are similar and are greater than that of the normal distribution. All the returns under investigation are clearly heavy tailed. Diesel and LTC are the only two positively skewed series. Overall, inspecting the histograms in Fig 3, we note that each participating return appears more or less symmetrically distributed around zero with the exception of the Propane.

Finally, we proceed to fit GARCH versions of the proposed distributions in Section 2 to the six data sets using the method of ML. Formally, suppose *x*_1_, *x*_2_, ⋯, *x*_*n*_ are independent observations, then the optimal parameters are the values maximizing the likelihood

$$\begin{array}{c} L(\Theta )=\prod_{i=1}^{n}f(x_{i};\Theta ), \end{array}$$

or in most cases due to computational convenience we use the log-likelihood as

$$\begin{array}{c} \log L(\Theta )=\sum_{i=1}^{n}\log f(x_{i};\Theta ), \end{array}$$

where **Θ** = (*θ*_1_…*θ_k_*)′ is the parameter vector. Consequently, the optimal estimates for **Θ** are $\hat{\Theta }={\left(\hat{\theta_{1}},\hat{\theta_{2}},\ldots ,\hat{\theta_{k}}\right)}^{{}^{\prime }}$. All our computations were performed using the standard Nelder-Mead optimization routine with optim command in R as provided by R Core Team [63].

Since the considered distributions are not nested, discrimination among them is performed using the Akaike information criterion (AIC) due to Akaike [64], the Bayesian information criterion (BIC) due to Schwarz [65], the corrected Akaike information criterion (AICc) due to Hurvich and Tsai [66], the Hannan-Quinn criterion (HQC) due to Hannan and Quinn [67], and the consistent Akaike information criterion (CAIC) due to Bozdogan [68]. Extensive discussion on these commonly used criteria is provided by Burnham and Anderson [69] and Fang [70]. Roughly speaking, the smaller the values of these criteria the better the fit.

## 4 Estimation results and discussion

The GARCH (1, 1) model with the six innovation distributions proposed in Section 2 was fitted to the data described in Section 3. The six innovation distributions do not allow for asymmetry. Also fitted is the GARCH (1, 1) model with the AST and GHYP distributions chosen as the innovation distributions. These two distributions allow for asymmetry of the volatility, which has been noted in the literature for cryptocurrency and energy data sets [37, 71, 72]. We have chosen GARCH (1, 1) as a baseline model, because it is the most simple and accessible model available in the R packages fGarch and rugarch for fitting GARCH type models. We fitted also GARCH models of higher orders, but they did not provide significantly better fits. The method of ML was used for fitting all of the models. For fitting the GARCH (1, 1) model with GHYP innovations, we used the rugarch package. For fitting the GARCH (1, 1) model with AST innovations, we used the VaRES package. The log-likelihood values and the values of two of the five selection criteria (AIC and BIC) for all the proposed distributions are provided in Table 2. The values of the three remaining selection criteria can be obtained from the authors. They led to the same conclusions. Table 2 also gives the differences in empirical and fitted estimates of kurtosis.

**Table 2:** Log-likelihood values, AIC values, BIC values and differences between empirical and fitted estimates of kurtosis for the GARCH(1, 1) model with the eight innovation distributions fitted to the specified daily log-returns (S&P500, DJI, Diesel, Propane, BTC and LTC).

Returns	Innovation distribution	−log*L*	AIC	BIC	*Δ* Kurtosis
S&P500	(14)	-14576.42	-29138.85	-29095.13	1.22
	(16)	-14557.67	-29103.34	-29065.87	1.54
	(18)	-14533.38	-29054.77	-29017.29	1.61
	(10)	-14220.60	-28429.21	-28391.73	1.85
	AST	-14178.9	-28351.80	-28333.06	2.08
	(12)	-14176.46	-28340.93	-28303.45	2.29
	GHYP	-14173.22	-28332.44	-28301.42	4.17
	(20)	-14170.14	-28330.28	-28299.05	4.89
DJI	(14)	-14983.02	-29952.04	-29908.32	0.64
	(16)	-14973.84	-29935.68	-29898.20	0.75
	(18)	-14945.96	-29879.93	-29842.45	1.85
	(10)	-14421.98	-28831.96	-28794.48	2.48
	AST	-14329.1	-28652.20	-28633.46	2.72
	(12)	-14323.70	-28635.40	-28597.93	3.28
	GHYP	-14311.13	-28608.26	-28545.48	5.46
	(20)	-14285.74	-28561.49	-28530.26	5.95
Diesel	(14)	-18882.19	-37750.38	-37704.24	0.78
	(16)	-17316.24	-34620.47	-34580.92	1.50
	(18)	-17109.01	-34206.01	-34166.46	2.10
	(10)	-16503.12	-32994.24	-32954.69	3.01
	AST	-16300.61	-32595.22	-32575.44	3.29
	(12)	-16286.97	-32561.95	-32522.39	3.78
	GHYP	-16281.32	-32548.64	-32500.09	4.54
	(20)	-16046.50	-32083.01	-32050.05	4.91
Propane	(14)	-18961.81	-37909.63	-37863.48	0.26
	(16)	-18168.30	-36324.59	-36285.04	1.49
	(18)	-16986.69	-33961.38	-33921.82	2.69
	(10)	-16595.60	-33179.20	-33139.64	3.78
	AST	-16511.43	-33016.86	-32997.08	3.80
	(12)	-16427.15	-32842.30	-32802.74	5.77
	GHYP	-16378.45	-32742.9	-32674.32	6.13
	(20)	-16319.42	-32628.84	-32595.88	6.67
BTC	(14)	-8976.20	-17938.40	-17896.61	0.90
	(16)	-8863.32	-17714.65	-17678.83	0.93
	(18)	-8599.89	-17187.78	-17151.97	1.83
	(10)	-8407.14	-16802.28	-16766.46	2.24
	AST	-8240.46	-16474.92	-16457.01	3.23
	(12)	-8032.90	-16053.79	-16017.98	3.62
	GHYP	-7832.78	-15651.56	-15611.87	4.01
	(20)	-7104.36	-14198.71	-14168.87	4.42
LTC	(14)	-5179.45	-10344.90	-10306.84	1.60
	(16)	-4755.62	-9499.24	-9466.62	1.70
	(18)	-4642.14	-9272.29	-9239.67	3.77
	(10)	-4456.49	-8900.99	-8868.37	4.13
	AST	-4402.01	-8798.02	-8781.71	4.14
	(12)	-4304.53	-8597.05	-8564.43	4.57
	GHYP	-4290.43	-8566.86	-8493.39	4.63
	(20)	-4182.78	-8355.55	-8328.37	6.90

According to the selection criteria and the kurtosis values in Table 2, the GARCH (1, 1) with compound generalized gamma innovations gives the best fit, the compound Burr innovations give the second best fit, the compound Fréchet innovations give the third best fit, the compound inverse gamma innovations give the fourth best fit, the AST innovations give the fifth best fit, the compound Lomax innovations give the sixth best fit and the GHYP innovations give the seventh best fit. The worst fit is given by the GARCH (1, 1) model with compound half normal innovations. These conclusions are the same for all the returns.

**Fig 4:**
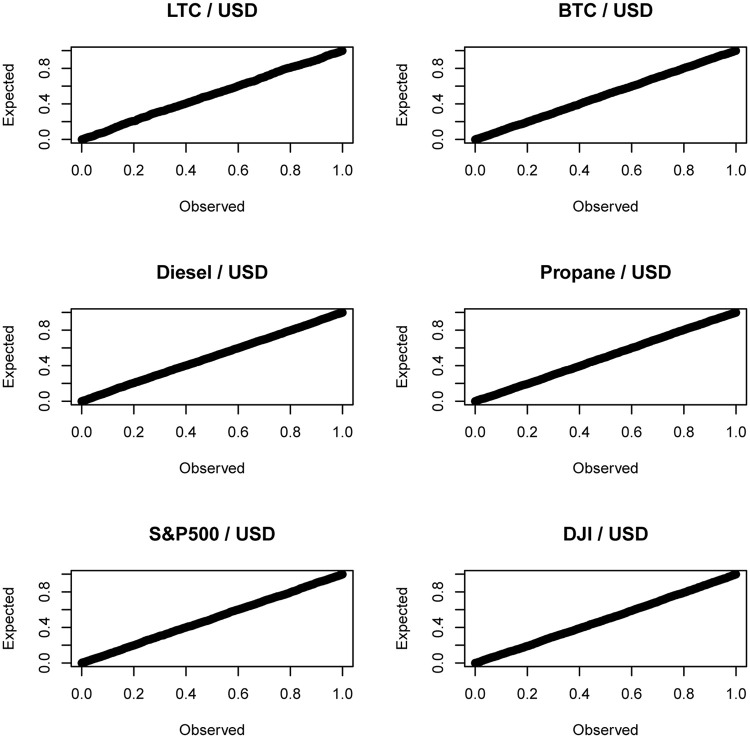
P-P plots of the standardized residuals of the GARCH(1, 1) model with innovations given by (14).

**Fig 5:**
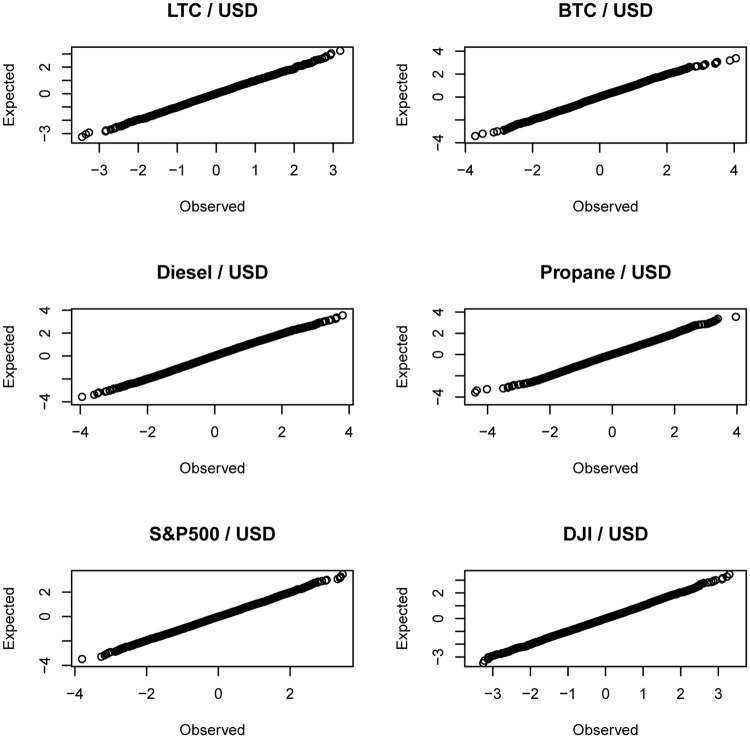
Q-Q plots of the standardized residuals of the GARCH(1, 1) model with innovations given by (14).

The probability plots of the standardized residuals for the best fitting GARCH (1, 1) model with compound generalized gamma innovations are shown in Fig 4. The corresponding quantile plots are shown in Fig 5. Both figures suggest that the fit of the model is adequate.

The *p*-values of Vuong [73]’s likelihood ratio test to see if the best fitting model is significantly better than the other seven models are given in Table 3. The *p*-values of Amisano and Giacomini [74]’s likelihood ratio test to see if the best fitting model is significantly better than the other seven models in the left and right tails are given in Table 4. The *p*-values in all these tables show that the GARCH (1, 1) model with compound generalized gamma innovations provides significantly better fits than all other models. Vuong [73]’s test was performed using the command vuongtest in the nonnest2 package. Amisano and Giacomini [74]’s test was performed using the code available in https://sites.google.com/site/gianniamisanowebsite/.

**Table 3:** *p*-values of Vuong 73’s likelihood ratio test comparing the GARCH(1, 1) with (14) versus the seven models.

Model	S&P500	DJI	Diesel	Propane	BTC	LTC
GARCH(1, 1) with (16)	0.019	0.018	0.016	0.020	0.014	0.015
GARCH(1, 1) with (18)	0.015	0.015	0.012	0.017	0.010	0.007
GARCH(1, 1) with (10)	0.008	0.010	0.010	0.013	0.008	0.005
GARCH(1, 1) with AST	0.008	0.010	0.009	0.013	0.007	0.003
GARCH(1, 1) with (12)	0.008	0.010	0.008	0.013	0.007	0.002
GARCH(1, 1) with GHYP	0.007	0.009	0.007	0.010	0.005	0.002
GARCH(1, 1) with (20)	0.001	0.005	0.006	0.005	0.004	0.001

**Table 4:** *p*-values of Amisano and Giacomini 74’s likelihood ratio test for the left (right in brackets) tails comparing the GARCH(1, 1) with (14) versus the seven models.

Model	S&P500	DJI	Diesel	Propane	BTC	LTC
GARCH(1, 1) with (16)	0.033	0.032	0.039	0.034	0.036	0.039
	(0.033)	(0.035)	(0.030)	(0.037)	(0.033)	(0.035)
GARCH(1, 1) with (18)	0.031	0.030	0.034	0.031	0.024	0.032
	(0.028)	(0.018)	(0.025)	(0.036)	(0.030)	(0.032)
GARCH(1, 1) with (10)	0.030	0.028	0.023	0.028	0.015	0.017
	(0.021)	(0.017)	(0.018)	(0.031)	(0.016)	(0.028)
GARCH(1, 1) with AST	0.016	0.021	0.020	0.027	0.013	0.015
	(0.021)	(0.015)	(0.017)	(0.025)	(0.012)	(0.025)
GARCH(1, 1) with (12)	0.013	0.020	0.005	0.026	0.012	0.014
	(0.020)	(0.014)	(0.016)	(0.022)	(0.010)	(0.024)
GARCH(1, 1) with GHYP	0.008	0.002	0.004	0.020	0.009	0.008
	(0.018)	(0.007)	(0.012)	(0.008)	(0.007)	(0.023)
GARCH(1, 1) with (20)	0.002	0.002	0.003	0.016	0.005	0.006
	(0.005)	(0.002)	(0.010)	(0.002)	(0.005)	(0.014)

Table 5 tests the significant difference between mean squared errors when the GARCH (1, 1) models were fitted to rolling windows of length 100 days and used to predict the 101th data value [75]. The GARCH (1, 1) with compound generalized gamma innovations is used as the baseline model. The R package fDMA was used to perform the tests. The *p*-values show that the GARCH (1, 1) model with compound generalized gamma innovations provides significantly better mean squared errors than all other models. These conclusions were the same when the widow length was taken to be 200, 300, …, 1000 days.

**Table 5:** *p*-values of Diebold and Mariano 75’s test comparing mean squared errors of the 101th day forecast for rolling windows of length 100 days for the GARCH(1, 1) with (14) versus the same for the seven models.

Model	S&P500	DJI	Diesel	Propane	BTC	LTC
GARCH(1, 1) with (20)	0.004	0.000	0.001	0.012	0.003	0.005
GARCH(1, 1) with GHYP	0.010	0.001	0.005	0.012	0.006	0.010
GARCH(1, 1) with (12)	0.018	0.017	0.024	0.021	0.012	0.013
GARCH(1, 1) with AST	0.023	0.017	0.025	0.036	0.015	0.016
GARCH(1, 1) with (10)	0.031	0.029	0.028	0.036	0.021	0.020
GARCH(1, 1) with (16)	0.038	0.033	0.029	0.040	0.028	0.023
GARCH(1, 1) with (14)	0.041	0.035	0.029	0.044	0.030	0.038
GARCH(1, 1) with (14)	0.044	0.048	0.046	0.046	0.035	0.049

Finally, Table 6 gives the *p*-values of three backtesting methods at 99 percent value-at-risk. In each triplet, the first is the *p*-value of Kupiec’s proportion of failures [76] test, the second is the *p*-value of Escanciano and Olmo [77]’s test, and the third is the *p*-value of peak over threshold’s method. For the last method, we used the evd package. The threshold was chosen by the mean residual plot which was drawn using the command mrlplot. As expected, the peak over threshold’s method gives the largest *p*-values. For the first two methods, the GARCH (1, 1) with compound generalized gamma innovations gives the largest *p*-values, the compound Burr innovations give the second largest *p*-values, the compound Fréchet innovations give the third largest *p*-values, the compound inverse gamma innovations give the fourth largest *p*-values, the AST innovations give the fifth largest *p*-values, the compound Lomax innovations give the sixth largest *p*-values, and the GHYP innovations give the seventh largest *p*-values. The smallest *p*-values with all of them below the 5 percent significance level are given by the GARCH (1, 1) model with compound half normal innovations. Some of the *p*-values for the GARCH (1, 1) model with GHYP innovations are also below the 5 percent level of significance. The remaining *p*-values are all above the 5 percent level of significance.

**Table 6:** *p*-values of Kupiec’s proportion of failures [76] test, Escanciano and Olmo [77]’s test and peak over threshold’s method for the eight models.

Model	S&P500	DJI	Diesel	Propane	BTC	LTC
GARCH(1, 1) with (14)	(0.37, 0.59, 0.62)	(0.58, 0.60, 0.64)	(0.59, 0.61, 0.63)	(0.51, 0.54, 0.60)	(0.55, 0.56, 0.59)	(0.58, 0.59, 0.65)
GARCH(1, 1) with (16)	(0.29, 0.41, 0.62)	(0.37, 0.49, 0.64)	(0.45, 0.49, 0.63)	(0.49, 0.52, 0.60)	(0.54, 0.55, 0.59)	(0.45, 0.46, 0.65)
GARCH(1, 1) with (18)	(0.23, 0.40, 0.62)	(0.28, 0.36, 0.64)	(0.41, 0.43, 0.63)	(0.21, 0.24, 0.60)	(0.45, 0.47, 0.59)	(0.40, 0.44, 0.65)
GARCH(1, 1) with (10)	(0.17, 0.25, 0.62)	(0.21, 0.29, 0.64)	(0.37, 0.39, 0.63)	(0.17, 0.19, 0.60)	(0.34, 0.39, 0.59)	(0.15, 0.22, 0.65)
GARCH(1, 1) with AST	(0.16, 0.23, 0.62)	(0.17, 0.25, 0.64)	(0.32, 0.28, 0.63)	(0.11, 0.19, 0.60)	(0.34, 0.39, 0.59)	(0.12, 0.20, 0.65)
GARCH(1, 1) with (12)	(0.16, 0.22, 0.62)	(0.16, 0.24, 0.64)	(0.15, 0.20, 0.63)	(0.09, 0.19, 0.60)	(0.32, 0.37, 0.59)	(0.11, 0.13, 0.65)
GARCH(1, 1) with GHYP	(0.11, 0.13, 0.62)	(0.12, 0.22, 0.64)	(0.09, 0.19, 0.63)	(0.02, 0.06, 0.60)	(0.25, 0.28, 0.59)	(0.06, 0.11, 0.65)
GARCH(1, 1) with (20)	(0.00, 0.03, 0.62)	(0.03, 0.04, 0.64)	(0.02, 0.05, 0.63)	(0.00, 0.02, 0.60)	(0.02, 0.03, 0.59)	(0.01, 0.04, 0.65)

## 5 Simulation study

In this section, we conduct a simulation study to assess the performance and accuracy of the ML estimators of the best fitting GARCH(1, 1) model with compound generalized gamma innovations. The following scheme was used:

1. simulate a sample of size *n* from the GARCH(1, 1) model with generalized gamma innovations;
2. estimate (*ν*, *β*, λ, *α*) and the three GARCH parameters;
3. repeat steps 1 and 2 ten thousand times;
4. hence, estimate the biases and the mean squared errors for the seven parameters;
5. repeat steps 1 to 4 for *n* = 20, 21, …, 500.

**Fig 6:**
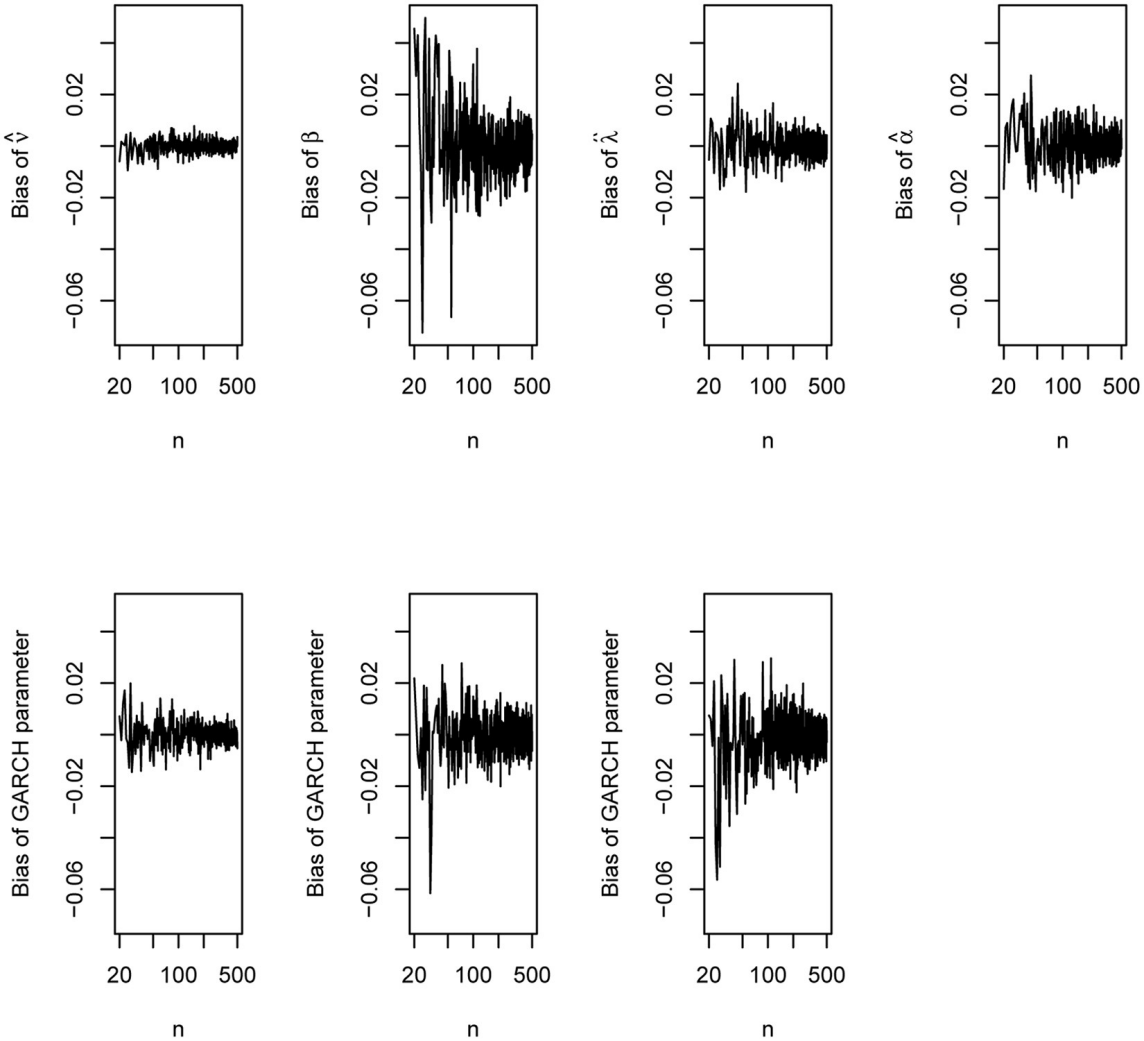
Biases of the parameter estimates of the GARCH(1, 1) model with innovations given by (14) based on the simulation study of Section 5.

**Fig 7:**
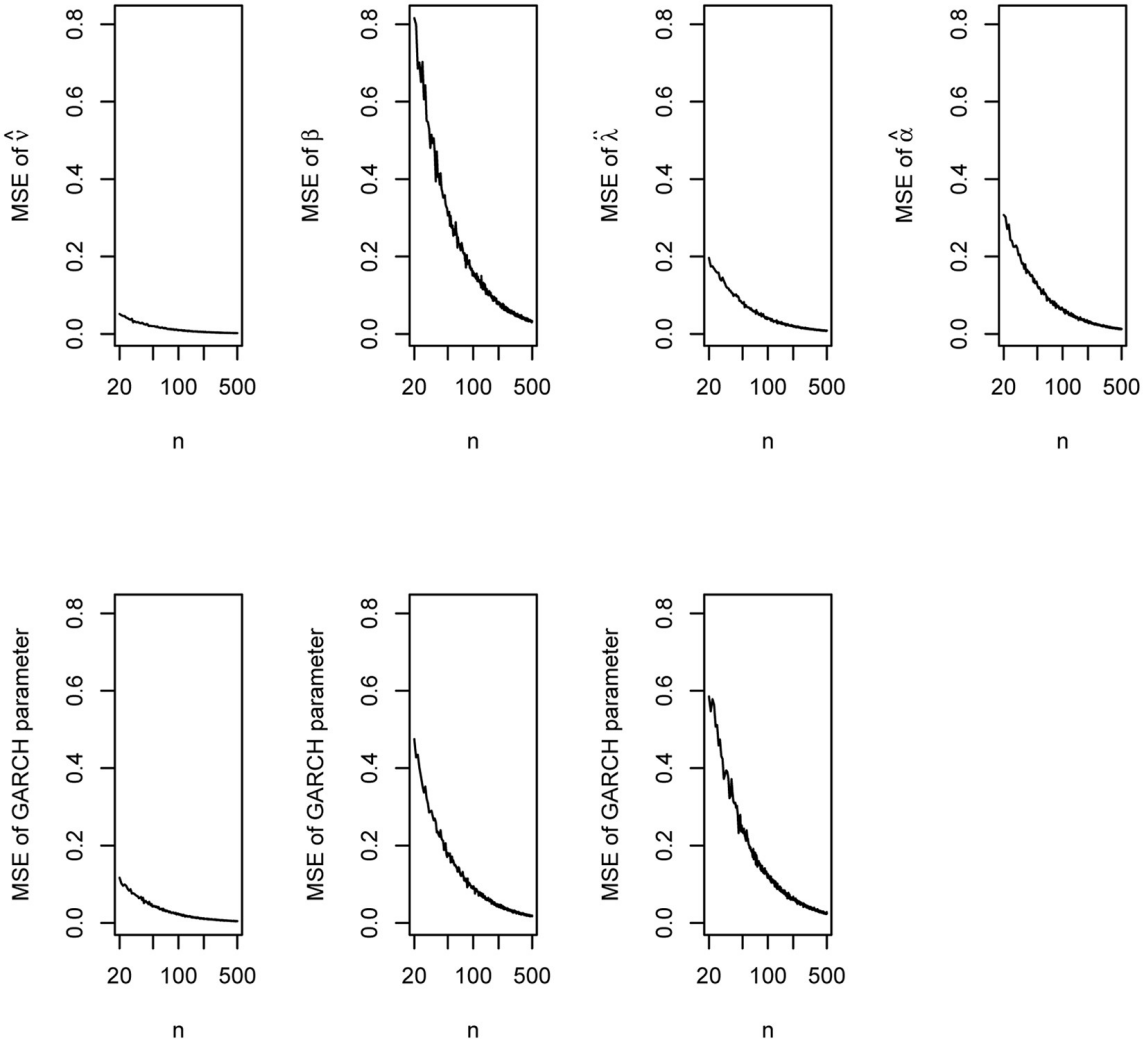
Mean squared errors of the parameter estimates of the GARCH(1, 1) model with innovations given by (14) based on the simulation study of Section 5.

The plots of the biases versus *n* are shown in Fig 6. The plots of the mean squared errors versus *n* are shown in Fig 7.

We can observe the following from the figures: the biases can be positive or negative but approach zero as *n* approaches 500; the biases appear largest for $\hat{\beta }$ and smallest for $\hat{\nu }$; the biases appear reasonably small at around *n* = 500; the mean squared errors gradually decrease with increasing *n*; the mean squared errors appear largest for $\hat{\beta }$ and smallest for $\hat{\nu }$; the mean squared errors appear reasonably small at around *n* = 500.

In the simulation scheme, we have taken the initial parameter values as the estimated values for S&P500 returns. The results were similar for a wide range of other initial values including the estimated values for the other five returns.

## 6 Conclusions

In this paper, based on the scale mixing of the Student’s *t* distribution, we have developed six new compound distributions. We have also derived their basic properties such as the PDF, CDF, moments and characteristic functions. With these distributions taken as innovations for the GARCH(1, 1) model, we have shown that all but one (respectively, two) of the six distributions perform better than the GARCH(1, 1) model with generalized hyperbolic (respectively, asymmetric Student’s *t*) innovations. The comparison was made in terms Akaike information criterion values, Bayesian information criterion values, consistent Akaike information criterion values, corrected Akaike information criterion values, Hannan-Quinn criterion values, *p*-values of Vuong [73]’s likelihood ratio test, *p*-values of Amisano and Giacomini [74]’s likelihood ratio test for the left tails, *p*-values of Amisano and Giacomini [74]’s likelihood ratio test for the right tails, mean squared errors of one-day ahead forecasts, and three backtesting methods.

In addition, we have performed a simulation study to examine the accuracy of the best fitting GARCH(1, 1) model with compound generalized gamma innovations. The accuracy was assessed in terms of biases and mean squared errors. Both decreased in magnitude when the sample size increased. Both appeared reasonably small when the sample size was as large as 500. The sample sizes of all six data sets considered are well above 500. The results showed that the GARCH (1, 1) model with compound generalized gamma innovations is valid and worth considering in the general financial context of risk exposure modelling.

Nearly all of the data sets we have considered have skewness close to zero. Hence, there is no need for the compound distributions in Section 2 to incorporate a skewness parameter. However, there are several ways that these distributions can be extended to incorporate skewness. A prominent approach is described in Theodossiou and Savva [78] and Savva and Theodossiou [79]. Another prominent approach is described in Fernández and Steel [5].

An extension of the paper is an analysis of the finiteness of the return distribution unconditional moments through the tail-index according to the “power law” literature; see Gabaix *et al.* [80], Ibragimov *et al.* [81] and references therein. This analysis could lead to a better understanding of the empirical results on the existence of the unconditional higher-order moments under the proposed distributions.

Further extensions to the GARCH time series frameworks could be also considered. However, framework of the Generalized Autoregressive Score (GAS) models of Creal *et al.* [82] and Harvey [83] is more intriguing. The GAS framework allows relatively straightforward introduction of the time-varying dynamics for any desired parameters and can enhance empirical performance of the suggested models further.

## Supporting information

S1 Dataset (XLSX)
